# A Novel 16-Genes Signature Scoring System as Prognostic Model to Evaluate Survival Risk in Patients with Glioblastoma

**DOI:** 10.3390/biomedicines10020317

**Published:** 2022-01-29

**Authors:** Zunpeng Yu, Manqing Du, Long Lu

**Affiliations:** School of Information Management, Wuhan University, Wuhan 430072, China; 2018101040011@whu.edu.cn (Z.Y.); 2020301041171@whu.edu.cn (M.D.)

**Keywords:** GBM, prognostic risk model, overall survival, survival-related genes

## Abstract

Previous studies have found that gene expression levels are associated with prognosis and some genes can be used to predict the survival risk of glioblastoma (GBM) patients. However, most of them just built the survival-related gene signature, and personal survival risk can be evaluated only in group. This study aimed to find the prognostic survival related genes of GBM, and construct survival risk prediction model, which can be used to evaluate survival risk by individual. We collected gene expression data and clinical information from the Gene Expression Omnibus (GEO) and The Cancer Genome Atlas (TCGA) databases. Cox regression analysis and LASSO-cox regression analysis were performed to get survival-related genes and establish the overall survival prediction model. The ROC curve and Kaplan Meier analysis were used to evaluate the prediction ability of the model in training set and two independent cohorts. We also analyzed the biological functions of survival-related genes by GO and KEGG enrichment analysis. We identified 99 genes associated with overall survival and selected 16 genes (*IGFBP2*, *GPRASP1*, *C1R*, *CHRM3*, *CLSTN2*, *NELL1*, *SEZ6L2*, *NMB*, *ICAM5*, *HPCAL4*, *SNAP91*, *PCSK1N*, *PGBD5*, *INA*, *UCHL1* and *LHX6*) to establish the survival risk prediction model. Multivariate Cox regression analysis indicted that the risk score could predict overall survival independent of age and gender. ROC analyses showed that our model was more robust than four existing signatures. The sixteen genes can also be potential transcriptional biomarkers and the model can assist doctors on clinical decision-making and personalized treatment of GBM patients.

## 1. Introduction

According to the 2016 WHO classification of tumors of the central nervous system, gliomas were classified into grade I–IV based on histopathology and genomics. Glioblastoma multiforme (GBM) is the highest malignant brain tumor as grade IV [1]. In the newest version of the classification of tumors of the central nervous system, numerous molecular changes with clinicopathologic utility that are important for the most accurate classification of CNS neoplasms were listed, such as *IDH*, *TERT* promoter, chromosomes 7/10, *EGFR*, which are important for glioblastoma [2]. Glioblastoma is the most common malignant primary brain tumor, accounting for 57.7% of all gliomas and 48.6% of all primary malignant central nervous system (CNS) tumors [3]. Although the treatment of glioblastoma has been greatly improved in diagnosis, surgery, traditional therapy such as chemoradiotherapy, and nursing, there are still efforts in immunotherapy, the overall prognosis is still discouraging, and the overall survival is still very poor due to the heterogeneity of glioblastoma [4]. For patients diagnosed with GBM, the median survival was about 12–15 months [5,6], and the 5-year survival rate was less than 10% [7].

With the development of high-throughput sequencing technology, many potential biomarkers related to the diagnosis and prognosis for treatment decision making of glioblastoma have been found, such as *MGMT* (O6 methylguanine DNA methyltransferase), H3F3A (H3 histone, family 3A), *IDH* (isocitrate dehydrogenase), *EGFR* (epidermal growth factor receptor), and *PTEN* (phosphatase and tensin homolog) [7,8,9,10,11]. The status of these biomarkers can provide a basis for individualized and targeted diagnosis and treatment management of patients.

Previous studies have shown that gene expression profiling can be used to classify glioma patients and identify patients with different overall survival and clinical characteristics [12,13,14,15,16,17]. GBM is divided into Proneural, Neural, Classical, and Mesenchymal subtypes by gene expression-based molecular classification, the result shows that different subtypes may require different therapeutic approaches [11,16]. Gene expression profiling can also be used to identify risk genes associated with survival and prognosis, and to assess the survival risk of patients. Zhang et al. identified 16 endoplasmic reticulum (ER) stress-related genes (*CYP2E1*, *SLN*, *BRCA1*, *CISD2*, *LRRK2*, *BMP2*, *MYH7*, *HSPB1*, *DNM1L*, *SHISA5*, *RNF185*, *RCN1*, *SPP1*, *RPN2*, *PDIA3* and *ATP2A2*), and established an ER stress risk model based on The Cancer Genome Atlas (TCGA) glioma database to reflect the immune characteristics and predict the prognosis of glioma patients [18]. Yin et al. analyzed the gene expression profiles and identified five novel biomarkers (*PTPRN*, *RGS14*, *G6PC3*, *IGFBP2* and *TIMP4*) that have potential in the prognosis prediction of GBM [19]. Wang et al. identified 14 autophagy-related genes (*MTMR14*, *LENG9*, *P4HB*, *TCIRG1*, *HSPA5*, *DRAM1*, *CTSD*, *S100A8*, *CCL2*, *MSTN*, *UBQLN4*, *PHYHIP*, *RRAGB* and *ZKSCAN3*) associated with the overall survival of patients with glioblastoma, and built a novel autophagy-related signature for the prediction of prognosis [20]. Cao et al. built a four-gene signature-derived (*OSMR*, *HOXC10*, *SCARA3* and *SLC39A10*) risk score model to predict the survival and treatment response of GBM patients [21]. Pan et al. identified two genes (*GRIA2* and *RYR3*) strongly associated with survival of GBM, and the two-gene signature was a robust prognostic model to predict GBM survival [22].

**Figure 1:**
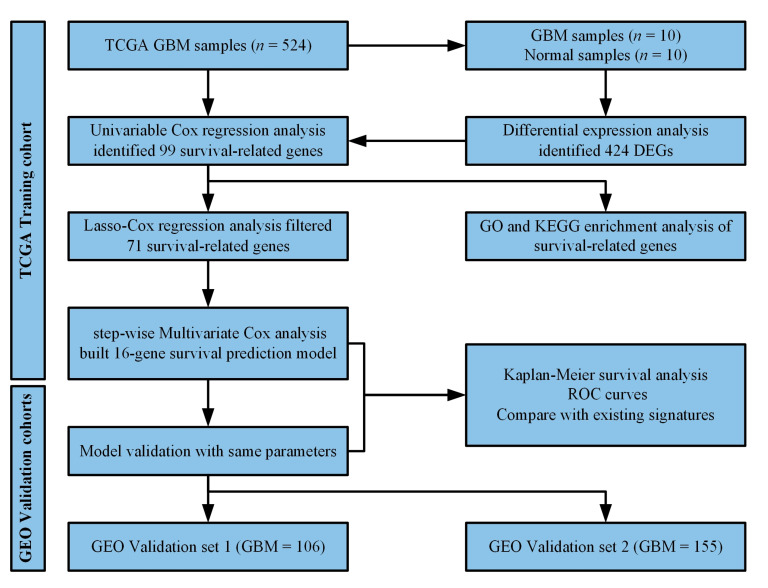
Schematic diagram of the study design and development of survival risk prediction model.

However, most of the studies just identified the survival-related genes and constructed a survival risk prediction model based on the prognostic signature, the model can generate a risk score for each patient, the risk score threshold divided patients into different risk groups. However, the risk score thresholds (usually the median risk score) are changed in different groups of patients collected from different institutions in these studies, which may be not convenient in clinical application. Based on public databases, we aim to explore novel prognostic biomarkers for survival prediction. By analyzing the genes expression profiles of GBM patients downloaded from Gene Expression Omnibus (GEO) and TCGA databases, we identified the prognostic genes and constructed a robust risk score model based on 16 genes, and we take the same risk score threshold in different groups. Therefore, our model can be used to predict survival risk for the individual patient with newly diagnosed glioblastoma. The workflow diagram of this study was shown in Figure 1.

## 2. Materials and Methods

### 2.1. TCGA Dataset

We downloaded the GBM gene expression array dataset (Affymetrix HT Human Genome U133 Array Plate Set, level 1) and clinical information from TCGA [10]. After removing duplicated samples, ten normal control samples and 524 disease samples were obtained, the clinical information included age, gender, survival time, survival status, and so on. TCGA data set served as the training set.

### 2.2. The GEO Dataset

We downloaded three raw data sets of gene expression from GEO: GSE4412 [15] and GSE4271 [16] are all generated by Affymetrix human genome U133A array Plate Set. After removing duplicate samples, GSE4412 contained 50 glioblastoma samples and GSE4271 contained 56 glioblastoma samples. We also downloaded the gene expression profiles of GSE16011 [17], which contained 155 glioblastoma samples, gene expression profiles were generated by Affymetrix Gene Chip Human Genome U133 Plus 2.0 Array Plate Set. GSE4412 and GSE4271 patients’ data were combined as validation set 1 and GSE16011 patients’ data were used as validation set 2.

All the raw data was preprocessed by the R package AFFY and the background correction and normalization were performed using robust multi-array analysis (RMA). SVA package [23] was used to remove batch effects.

The clinical information of all patients is shown in Table S1.

### 2.3. Differential Expression Analysis

There are 10 normal samples served as the control group, and 10 tumor samples were randomly selected from TCGA training set served as the GBM group. The differentially expressed genes (DEGs) between the GBM group and the control group were analyzed by the limma software package [24] in R (version 4.0.5). Generally, the genes whose |log(FC)| > 1 and adjusted *p*-value < 0.05 is considered to be statistically significant DEGs [25]. In this study, the genes were considered as DEGs by the standards of whose adjusted *p*-value < 0.01 and |log(FC)| > 2.5. R package pheatmap [26] was used to draw heatmap while ggplot2 (https://cran.r-project.org/web/packages/ggplot2/, accessed on 20 August 2021) was used to draw volcano map.

### 2.4. Identify Genes Associated with Survival

Univariable Cox regression analysis was performed with differentially expressed genes in the survival R software package (https://cran.r-project.org/web/views/Survival.html, accessed on 20 August 2021) in order to analyze the correlation between each gene and overall survival. Genes with log-rank *p* < 0.05 were considered to be associated with overall survival [27]. The survival-related genes were further filtered by the Lasso-Cox regression model [28,29,30] in the glmnet R package (https://cran.r-project.org/web/packages/glmnet/index.html, accessed on 20 August 2021) to reduce the dimensionality of inputted variables.

### 2.5. Construction of Survival Risk Model

In order to optimize the model, step-wise Multivariate Cox analysis was used to further filter the survival risk related genes and construct the survival risk model by the “survival”, “survminer” packages:

Risk score = h_0_ × exp(gene_1_ × coefficient_1_ + gene_2_ × coefficient_2_ + gene_i_ × coefficient_i_) (1)

Here, h_0_ is a constant, gene_i_ is the gene expression level. We assigned risk scores to each sample in the training cohort according to these gene expression levels. The patients were divided into low-risk group and high-risk group by the median risk score. The prognostic value of the model was evaluated by Kaplan-Meier survival analysis, Log-rank test, and time-dependent receiver operating characteristic (ROC) curve with “survival”, “survminer”, and “timeROC” packages in R.

### 2.6. Risk Model Validation

The validation set 1 and validation set 2 were employed to validate the robustness of diagnostic accuracy on overall survival by the prediction model. ROC curve and Kaplan Meier analysis were used to verify the prognostic value of GBM patients. *p*-value < 0.05 was considered statistically significant. Multivariate Cox analysis was used to validate whether the risk score was an independent risk factor for GBM survival.

### 2.7. Compare with Existing Signatures

As mentioned at first, lots of genes have been found related to overall survival and could be used to predict the prognosis of patients with GBM. We compared the prognostic capability of our model with signatures reported in other studies, including the five-gene signature (*PTPRN*, *RGS14*, *G6PC3*, *IGFBP2* and *TIMP4*) screened by Yin et al. [19], the two-gene signature (*GRIA2* and *RYR3*) screened by Pan et al. [22], the five-gene signature (*DES*, *RANBP17*, *CLEC5A*, *HOXC11* and *POSTN*) screened by Wang et al. [25], the four-gene signature (*LHX2*, *MEOX2*, *SNAI2* and *ZNF22*) derived by Cheng et al. [31].

For each signature, we first conducted Multivariate Cox regression analysis and built the prognostic model with its genes in TGCA cohort. Then according to the median risk score, the patients were divided into low- and high-risk groups in TGCA cohort and two validation cohorts. Kaplan-Meier analysis and ROC curves were performed to evaluate the prognostic power of each model.

### 2.8. GO and KEGG Enrichment Analysis of Survival-Related Genes

In order to identify potential molecular biomechanisms of genes related to survival, we conducted functional enrichment analyses including GO (Gene Ontology) and KEGG (Kyoto Encyclopedia of Genes and Genomes) pathways analysis and visualized the results by “ggplot2” package.

## 3. Results

### 3.1. Differentially Expressed Genes Can Clearly Distinguish GBM Patients from Normal Samples

**Figure 2:**
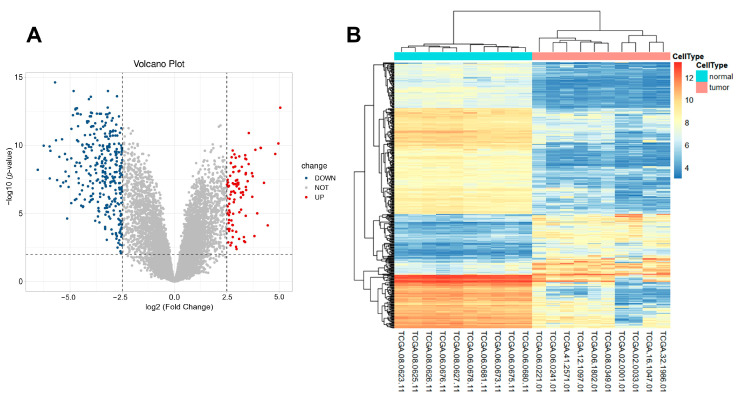
Identification of DEGs between GBM and normal brain tissues. (**A**) Volcano plots of differentially expressed genes showing the log (Fold Change) of mRNA in GBM compared to normal brain tissues, and the corresponding–log10 (adjusted *p*-value). Genes with adjusted *p*-value below 0.01 and fold change above 2.5 (below −2.5) were marked with red (blue) dots; (**B**) Heatmap of differentially expressed genes. GBM samples can be clearly distinguished from normal samples according to differentially expressed genes that are from (**A**). The color bar means the expression level of differentially expressed genes.

In total, 424 differentially expressed genes were identified by differential expression analysis of 10 normal brain tissue samples and 10 GBM tumor samples. Among these genes, 327 were down regulated and 97 were up regulated in GBM tumor tissue (Figure 2A). These differentially expressed genes can clearly distinguish GBM samples from normal samples, tumor samples were tightly clustered together and notably separated from normal samples (Figure 2B). The 424 differently expressed genes were listed in Table S2.

### 3.2. 99 DEGs Were Significantly Related to Survival

**Figure 3:**
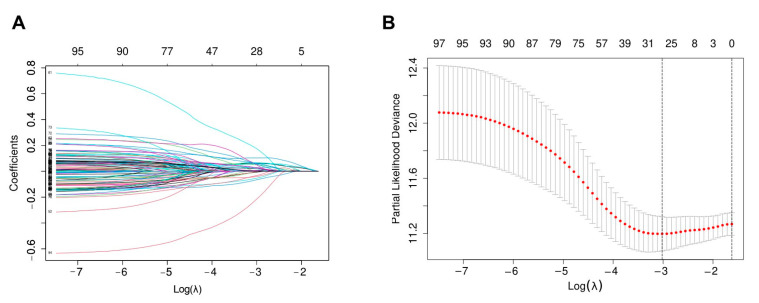
Lasso-cox gene screening process. (**A**) LASSO coefficient profiles of the 99 genes in TCGA GBM cohort; (**B**) Selection of the best parameter (lambda) in the LASSO-Cox model.

Univariate Cox regression analysis was performed to identify the overall survival related genes in the training set. 99 genes were identified as potential survival-related biomarkers of GBM patients (*p* < 0.05) (Table S3). LASSO Cox regression analysis was performed to further analyze these 99 survival-related genes to avoid overfitting [32,33], genes were filtered by 10-fold cross-validation with a maximum of 1000 iterations, and 28 genes were screened for subsequent analysis (Figure 3).

### 3.3. The Risk Score of Sixteen-Gene Model Were Strongly Associated with Overall Survival of GBM Patients

**Figure 4:**
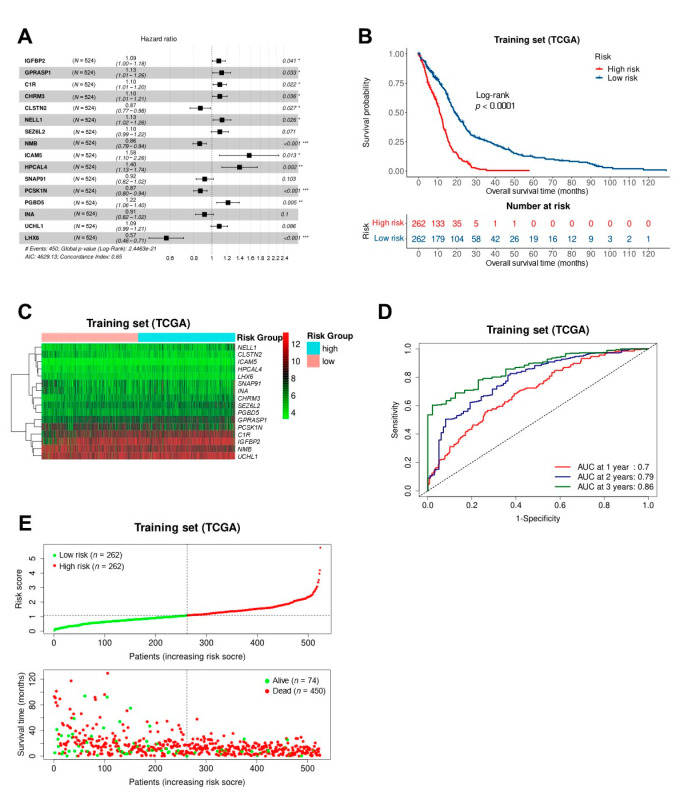
Establishment of the survival risk model. (**A**) 16 genes were eventually selected to establish prognostic model. ***: *p* < 0.001, **: *p* < 0.01, *: *p* < 0.05; (**B**) Kaplan-Meier curve of overall survival in high- and low-risk group of the GBM patients in the training cohort; (**C**) Heatmap of 16 genes expression patterns; (**D**) ROC curves of the model for overall survival at 1-, 2-, and 3- years; (**E**) The distribution risk scores in the TCGA cohort.

Furthermore, multivariate Cox analysis narrowed the list down further to 16 survival related genes and we constructed the survival risk model with these genes, which can be used to predict the overall survival risk of patients with GBM (Figure 4A). Of these genes, *CLSTN2*, *NMB*, *SNAP91*, *PCSK1N*, *INA*, and *LHX6* were favorable prognostic factors for glioblastoma survival, whose risk ratio (HR) < 1, the regression coefficient < 0. *IGFBP2*, *GPRASP1*, *C1R*, *CHRM3*, *NELL1*, *SEZ6L2*, *ICAM5*, *HPCAL4*, *PGBD5,* and *UCHL1* were risk factors, whose HR > 1, the regression coefficient > 0. The risk score of each patient was generated as follows: 0.04701 × exp(0.0842 × *IGFBP2* + 0.1187 × *GPRASP1* + 0.0962 × *C1R* + 0.0965 × *CHRM3* − 0.1409 × *CLSTN2* + 0.1231 × *NELL1* + 0.0965 × *SEZ6L2* − 0.149 × *NMB* + 0.4547 × *ICAM5* + 0.3367 × *HPCAL4* − 0.0876 × *SNAP91* − 0.1406 × *PCSK1N* + 0.1966 × *PGBD5* − 0.0901 × *INA* + 0.0897 × *UCHL1* − 0.5555 × *LHX6*) (Table 1).

The patients were divided into high-risk group and low-risk group according to the median risk score. Kaplan-Meier survival analysis and log-rank test of high-risk and low-risk GBM patients showed that there was a significant difference in the overall survival between the two groups (*p* < 0.0001). Patients in the low-risk group had a better prognosis, that was to say, the patients had longer overall survival, the median overall survival of high and low risk groups respectively were 10.23 and 16.12 months (Figure 4B). The time-dependent ROC curves were generated to assess the ability to discriminate the prognostic risk of the model. The areas under the curve (AUC) of predicting 1-, 2-, and 3-years OS in TCGA dataset were 0.7, 0.79, and 0.86, respectively (Figure 4D). The expression patterns of 16 genes in high and low risk groups of TCGA cohort were shown in Figure 4C. With the increase of risk score, the expression level of *CLSTN2*, *NMB*, *SNAP91*, *PCSK1N*, *INA*, and *LHX6* were lower and lower, and the expression level of the other genes were higher and higher. The distribution of risk scores and survival information for patients in high and low risk groups were shown in Figure 4E, higher risk score patients have a shorter survival time.

### 3.4. The Model Was Sufficient and Effective in Predicting Overall Survival in GBM External Verification

**Figure 5:**
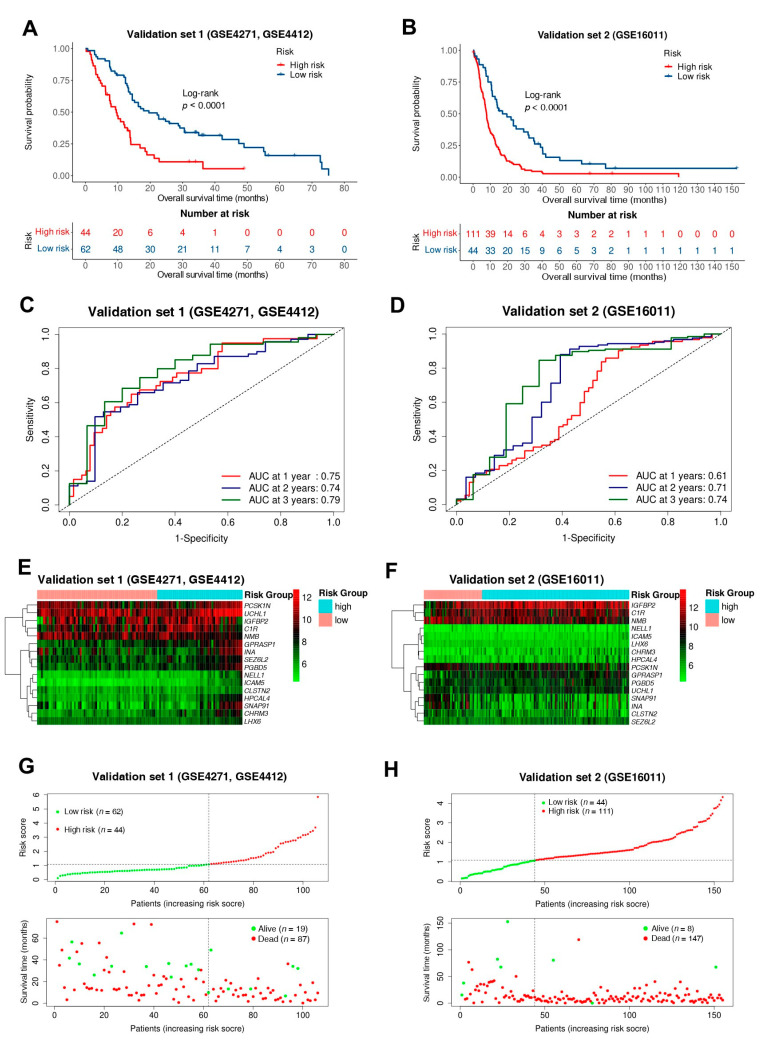
Validation and evaluation of the effectiveness of the survival risk model. (**A**,**B**) Kaplan-Meier survival analysis curves of validation set 1, 2; (**C**,**D**) Time-dependent ROC curves of validation set 1, 2 for the first, second, and third years; (**E**,**F**) Heatmap of 16 gene expression profiles in validation set 1, 2. Red parts indicate higher expression and green parts indicate lower expression; (**G**,**H**) Risk scores distribution and survival status scatter plots of patients in validation set 1, 2.

Two external validation sets were then used to evaluate the prediction efficiency of the model. Using the same model and parameters, patients in the validation sets were also divided into high-risk and low-risk groups. Similar to the training set, the overall survival of the high-risk group was significantly shorter than that of the low-risk group (*p* < 0.05), the median overall survival of high and low risk groups in validation set 1 respectively are 9.42 and 18.78 months, the median overall survival of high and low risk groups in validation set 2 respectively were 7.79 and 16.18 months (Figure 5A,B). The AUC values of 1-, 2-, 3-years overall survival prediction in validation sets, respectively, are 0.75, 0.74, 0.79 and 0.61, 0.71, 0.74 (Figure 5C,D). The expression patterns of 16 genes in verification set 1 and 2 were shown in Figure 5E,F, the expression patterns with the increase of risk score of these genes in validation cohorts have similar trends as in the training set. Figure 5G,H showed the distribution of risk scores and survival information of patients in the validation sets, it also shows that the higher the risk score, the shorter the patient’s survival. These results indicated the accuracy of the survival risk model constructed by 16 genes in predicting the prognosis of patients with glioblastoma.

To further verify the independence of the prognostic model, Univariate and Multivariate Cox regression analyses were performed. The result showed that the prognosis risk score was significantly associated with overall survival, independent of clinical factors including age and gender (Table 2).

### 3.5. The Sixteen-Gene Model Was More Robust and Effective Compaired with Four Existing-Survival-Related Gene Signatures

**Figure 6:**
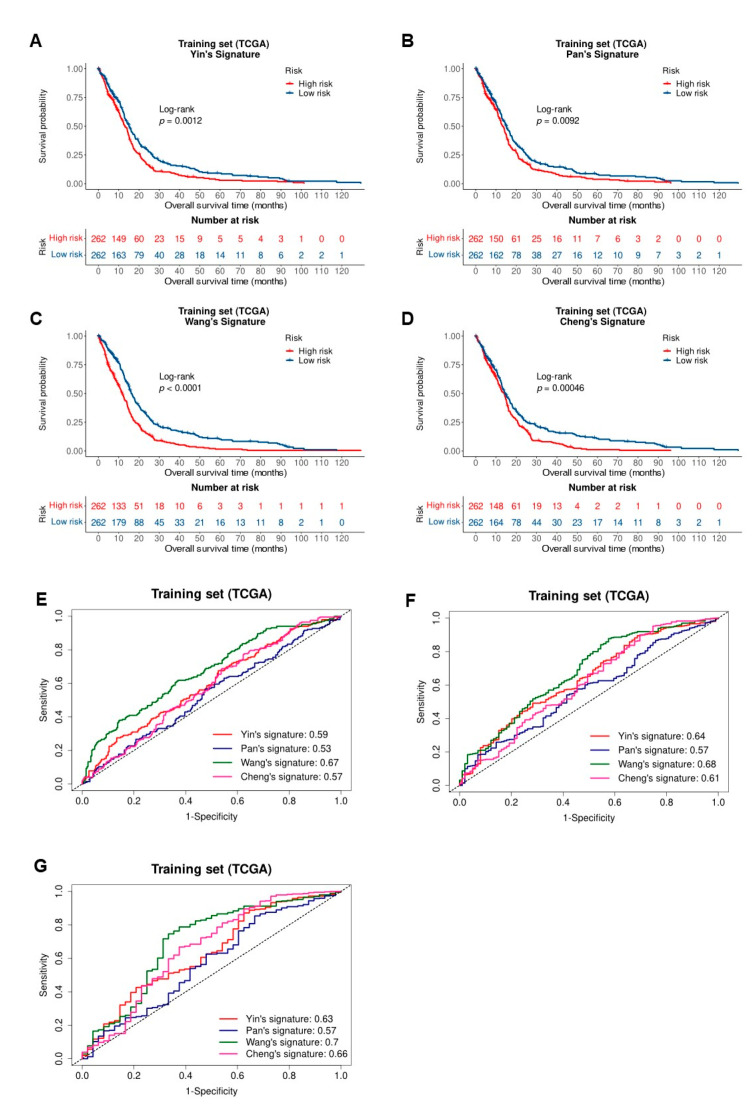
Comparison of ability for survival prediction by the sixteen-gene signature and four published prognostic signatures in the training cohort. (**A**–**D**) Kaplan-Meier analysis of four published signatures for the overall survival of glioblastoma patients in training cohort; (**E**–**G**) The ROC curves of different models for predicting 1-, 2-, 3-year overall survival in the training cohort.

We also built the survival prediction model with four existing survival-related signatures We evaluated the ability of these models for predicting the survival risk of GBM patients in the three cohorts. The result showed that Yin’s signature also was robust in predicting the prognosis risk of patients with GBM (log-rank test *p* < 0.05 in all three cohorts; Figure 6A and Figure S1A,H). The smaller the *p*-value, the greater the difference in survival between the two groups. The results of Kaplan-Meier survival analysis, ROC curves, and AUC values of different models for predicting 1-, 2-, 3-years overall survival in training cohort were shown in Figure 6A–G. The results of Kaplan-Meier survival analysis, ROC curves, and AUC values of these models to predict 1-, 2-, 3-years overall survival in validation sets were shown in Figure S1. Although the models can divide GBM patients into low and high-risk groups, the difference in survival between the two groups was mostly insignificant. The performance of our model and the models of the existing four signatures was summarized in Table 3. As shown in the table, most of the AUC values of our model were bigger than the existing models in the three cohorts. The performance of these four models for survival risk prediction was very poor in the validation set 1, the *p*-values of Pan’s, Wang’s and Cheng’s signature are greater than 0.05, and the AUC values of the four models were only about 0.5–0.6 for predicting 1-, 2-, 3-years overall survival. And the AUC values of our model for predicting 1-, 2-, 3-years overall survival in three cohorts were more stable. These results demonstrated that our model was more robust and powerful to predict overall survival than the four existing signatures.

### 3.6. The Risk Scores Were Association with Some Critical Clinicopathological Parameters

**Figure 7:**
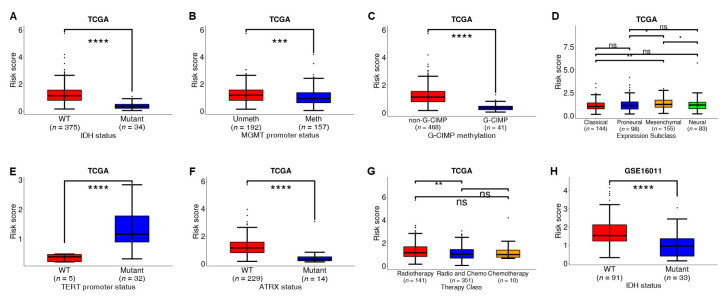
Correlation between risk score and clinicopathology. (**A**,**H**) Comparison of risk scores in *IDH*-WT GBM and *IDH*-Mutant GBM in TCGA cohort and GSE16011 cohort; (**B**) Comparison of risk scores in *MGMT*-promoter- methylation, and *MGMT*-promoter- unmethylation GBM in the TCGA cohort; (**C**) Comparison of risk scores in G-CIMP and non-G-CIMP methylation GBM in the TCGA cohort; (**D**) Comparison of risk scores in Proneural, Neural, Classical, and Mesenchymal GBM in the TCGA cohort; (**E**) Comparison of risk scores in *TERT*-WT and *TERT*-Mutant GBM in TCGA cohort; (**F**) Comparison of risk scores in *ATRX*-WT and *ATRX*-Mutant GBM in the TCGA cohort; (**G**) Comparison of risk scores for therapeutic modalities in the TCGA cohort (only radiotherapy, radiotherapy and chemotherapy, only chemotherapy). ****: *p* ≤ 0.0001, ***: *p* ≤ 0.001, **: *p* ≤ 0.01, *: *p* ≤ 0.05, ns: *p* > 0.05.

In training set and validation set 2, the correlation between risk score and some important clinicopathological features (therapeutic modalities, key molecular biomarkers, etc.) was analyzed. The results showed that there was a statistically significant difference in risk score between GBM patients with *IDH* wild type and *IDH* mutation type, GBM patients with *IDH* wild type have higher risk scores both in TCGA cohort and GSE16011 cohort (Figure 7A,H), which was also demonstrated in previous studies [20,21,34]. The higher risk score was associated with *MGMT* promoter unmethylation subtype, non-methylated subtype, Mesenchymal subtype, *TERT* mutation type, *ATRX* wild type, and radiotherapy alone (Figure 7B–G), and high-risk score means poor prognosis, they were all risk factors for survival. All these results indicate that a high-risk score was associated with short survival.

To confirm and further convince the independent of the prognostic efficacy of the risk model, the clinical-pathological factors such as therapeutic modalities, key genes status, and expression subtypes, GBM patients were stratified by these factors, and Kaplan Meier survival analysis was performed in the training cohort and validation set 2 cohort. Kaplan Meier survival analysis was also performed for high-risk and low-risk groups of GBM patients combined with clinical parameters. As shown in Figures S2 and S3, there were generally and significantly differences in overall survival between high-risk and low-risk groups of patients among stratified GBM patients by clinical-pathological factors. These results suggest that the model can distinguish the high-risk subgroup and the low-risk subgroup in each clinical subtype, the risk score can effectively predict the survival risk of patients with glioblastoma. Univariate and Multivariate Cox regression analyses were also performed with patients who having the clinical-pathological and molecular characteristics information in TCGA training set (206 samples) and validation set 2 (92 samples). The results showed that the prognosis risk score of our model was significantly associated with overall survival, independent of clinical and molecular characteristics such as *IDH* status, *MGMT* promoter status (Table S4). The analysis also shows that some molecular markers and clinical information also have the ability of survival risk assessment, such as Karnofsky Performance Status (KPS) score.

### 3.7. The Biological Pathway of Survival-Related Genes Involved In

**Figure 8:**
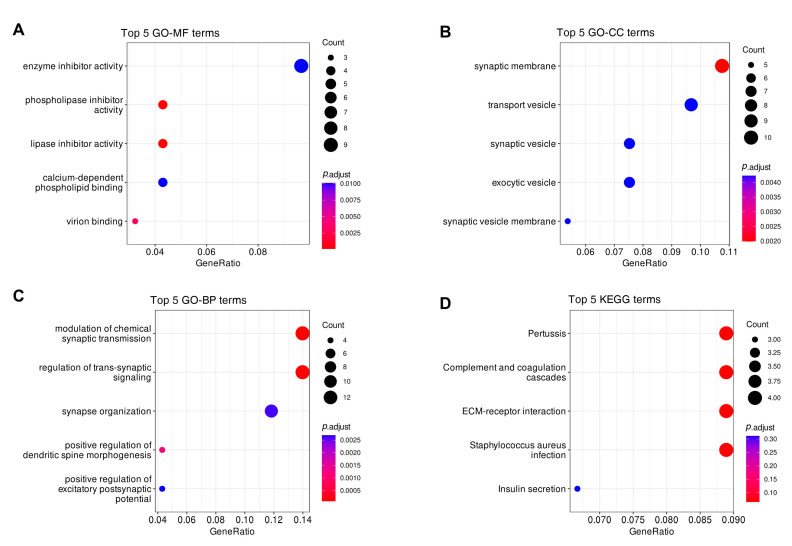
The Gene Ontology (GO) and Kyoto Encyclopedia of Genes and Genomes (KEGG) pathway enrichment analysis of genes associated with overall survival in GBM patients. (**A**) The top 5 statistically significant enriched terms in molecular function; (**B**) The top 5 statistically significant enriched terms in cellular components; (**C**) The top 5 statistically significant enriched terms in biological process; (**D**) The top 5 enriched KEGG pathways (Some are not statistically significant).

We constructed the model using 16 survival-related genes, due to the number of these 16 genes being too small to enrich for significant pathways, we used all 99 survival-related genes for pathway analysis. Functional enrichment analysis of 99 survival-related genes was conducted, and the results are shown in Figure 8. For molecular functions (MF), the major enriched GO terms were enzyme inhibitor activity, phospholipase inhibitor activity, ion channel binding, etc. (Figure 8A). For cellular components (CC), the genes were mainly enriched in the synaptic membrane, transport vesicle, synaptic vesicle, etc. (Figure 8B). For the biological processes (BP), the major enriched GO terms were the modulation of chemical synaptic transmission, regulation of trans-synaptic signaling, synapse organization, etc. (Figure 8C). According to KEGG analysis, these genes are mainly enriched in Pertussis, Complement and coagulation cascades, ECM receptor interaction, and many other pathways (Figure 8D).

In 16 survival-related genes, *GPRASP1*, *CHRM3*, *CLSTN2*, *NELL1*, *SEZ6L2*, *ICAM5*, *HPCAL4*, *SNAP91*, *PCSK1N*, *PGBD5*, *INA*, *UCHL1* and *LHX6* were down regulated, *IGFBP2*, *C1R* and *NMB* were up regulated compared with normal tissue samples. *GPRASP1* has been found overexpressed in brain, pancreatic, and breast cancers as compared to their respective normal tissues, and it may be a biomarker for early-stage cancer [35]. *CHRM3* may play a vital role in the deficits of the thalamus–cortical connectivities and is associated with schizophrenia dysfunction [36]. Overexpression of *CHRM3* or activation of *CHRM3* by carbachol promoted cell proliferation, migration, and castration resistance in prostate cancer [37]. *CLSTN2* is associated with episodic memory and late-onset Alzheimer’s disease [38,39]. *NELL1* has been found to be associated with a variety of tumors, which may inhibit the progress of cancer. That makes it a promising candidate biomarker of tumor suppressor genes and a potential target for tumor therapy [40,41,42,43,44]. *SEZ6L2* is highly expressed in colorectal cancer and high expression means a poor prognosis [45]. It also is an important regulator of drug-resistant cells and tumor spheroid cells in lung adenocarcinoma [46]. *ICAM5* is an intercellular adhesion molecule and may play a role in tumorigenesis and perineural invasion, most likely through P13K/Akt signaling pathway [47]. *ICAM-5* regulates T cell activity and participates in the immune privilege of the brain. It may be a useful anti-inflammatory agent for the treatment of various inflammatory brain diseases [48]. Some studies have found that *HPCAL4* may be the core gene of GBM and a potential therapeutic target [49]. It also has been found can affect many cellular processes, such as homeostasis, learning and memory, cancer, and pain [50]. *SNAP91* is associated with Alzheimer’s disease [51], schizophrenia [52], Parkinson’s disease [53] and colorectal cancer [54]. *PCSK1N* has an association with various neurodegenerative diseases, widely expressed in neurons throughout the brain [55,56]. *PGBD5* which encodes an active DNA transposase was highly expressed in various childhood and adult solid tumors [57]. The *PGBD5* DNA transposase confers a synthetic dependency on DNA damage repair and signaling, and expression of *PGBD5* induces DNA damage, which requires both DNA damage repair and DNA damage signaling, resulting in apoptosis if impaired by their selective inhibitors [58]. It also has been found to be associated with frontotemporal dementia [59]. *INA* is a strong prognostic factor in gliomas [60]. *UCHL1* may play an important role in maintaining axonal function after cerebral ischemia [61]. *UCHL1* is also associated with many types of cancers including lung, colorectal, breast cancer, and pancreatic, which can promote tumor progression [62,63]. *LHX6* is associated with many kinds of cancers and can inhibit the proliferation, invasion, and migration of breast cancer cells by modulating the PI3K/Akt/mTOR and Wnt/β-catenin signaling pathways [64,65,66]. *LHX6* plays a tumor-suppressing role in MC-LR-induced liver cancer through the Wnt/β-catenin and P53 pathways [67]. *IGFBP2* plays an essential role in cognitive development during early life, it is a potential target for learning and memory impairment therapies and neurodegenerative diseases [68]. *IGFBP2* is overexpressed and promotes many key oncogenic processes, including epithelial-to-mesenchymal transition, cellular migration, invasion, angiogenesis, stemness, transcriptional activation, and epigenetic programming in many solid tumors [69]. *IGFBP2* promotes tumor progression in pancreatic ductal adenocarcinoma through the STAT3 pathway [70]. *C1R* is up-regulated in cutaneous squamous cell carcinoma [71]. DNA-methylation of *C1R* is significantly correlated with overall survival in acute myeloid leukemia [72]. *NMB* is expressed in non-tumorigenic cells, but its expression is low to undetectable in malignant cells [73], and several studies have shown that *NMB* may act as a tumor suppressor [74,75,76,77].

## 4. Discussion

Glioblastoma is a kind of central nervous system tumor with a poor prognosis. Combined with clinical and overall survival information, we analyzed in-depth the gene expression data of GBM patients and constructed the survival risk prediction model. We found some survival-related genes and analyzed their molecular biomechanisms. These genes can be used as potential biomarkers in diagnosis and treatment. The results and our model can support clinical decision-making for the diagnosis and prognosis of the disease, so as to provide effective treatment and obtain better prognostic outcomes.

In this study, a total of 424 differentially expressed genes were obtained between GBM and normal brain tissue. Univariate Cox analysis showed that 99 genes were associated with overall survival. The survival-related genes were selected for further analysis. In the training set, 16 genes related to overall survival were screened by LASSO-Cox regression analysis and multiple Cox regression analysis. We found that the expression levels of *CLSTN2*, *NMB*, *SNAP91*, *PCSK1N*, *INA* and *LHX6* were positively correlated with the overall survival of glioblastoma, while the expression levels of *IGFBP2*, *GPRASP1*, *C1R*, *CHRM3*, *NELL1*, *SEZ6L2*, *ICAM5*, *HPCAL4*, *PGBD5* and *UCHL1* were negatively correlated with overall survival. All these genes have been found to be associated with diseases or cancers. It reflects from the side the biological significance of the survival-related genes we selected and the effectiveness of our model.

Based on these 16 genes, we built a prognostic survival risk model. The patients were divided into low-risk group and high-risk group by the median risk score. Two external datasets verified the effectiveness of the model, the results show that the model was more robust and can effectively predict the survival risk of patients. Because we took the same risk score threshold in different groups, we didn’t have to adjust the risk cutoff value of high and low risk groups in different GBM patients’ datasets, so our model can be used to predict survival risk for a new individual patient of glioblastoma.

We also found that GBM patients with *IDH* wild type, *MGMT* promoter unmethylation, Mesenchymal subtype, *TERT* mutation have higher risk scores, which means these are all negative factors. The model can distinguish the high-risk subgroup and the low-risk subgroup in each clinical subtype.

Our model was more robust and effective compared with the existing four gene-related signatures, maybe because we used three steps to selected survival-related genes and constructed the model, trying to search for survival-related gene combinations as best as possible and reduce the redundancy in genes to construct the optimal model.

There are some limitations to our work. First of all, our differential expression analysis only contained a very limited number of normal samples. The number of genes included in the study is only more than 12,000, which may overlook some potential mRNAs. Secondly, this study did not explore the protein expression of these 16 genes and their prognostic effects. Thirdly, the reliability of the scoring model needs further clinical and experimental verification. Fourthly, the genes expression data we used were only generated by Affymetrix human genome U133A array Plate Set and Affymetrix Gene Chip Human Genome U133 Plus 2.0 Array Plate Set, our model may not be suitable for data generated by other chips, because the data generated by different types of chips may be very different.

## Figures and Tables

**Table 1:** The details of the 16 genes used in the prognostic prediction model.

Gene Name	Coef.	HR	95% CI	*p*-Value
*IGFBP2*	0.0842	1.0878	1.0036–1.1790	0.0405
*GPRASP1*	0.1187	1.1260	1.0099–1.2555	0.0325
*C1R*	0.0962	1.1010	1.0142–1.1951	0.0216
*CHRM3*	0.0965	1.1013	1.0063–1.2054	0.0361
*CLSTN2*	−0.1409	0.8686	0.7665–0.9842	0.0272
*NELL1*	0.1231	1.1310	1.0151–1.2600	0.0256
*SEZ6L2*	0.0965	1.1013	0.9918–1.2230	0.0710
*NMB*	−0.1490	0.8616	0.7935–0.9354	0.0004
*ICAM5*	0.4547	1.5757	1.0990–2.2592	0.0134
*HPCAL4*	0.3367	1.4004	1.1277–1.7389	0.0023
*SNAP91*	−0.0876	0.9162	0.8245–1.0180	0.1034
*PCSK1N*	−0.1406	0.8688	0.8007–0.9427	0.0007
*PGBD5*	0.1966	1.2173	1.0611–1.3966	0.0050
*INA*	−0.0901	0.9139	0.8208–1.0175	0.1003
*UCHL1*	0.0897	1.0938	0.9874–1.2117	0.0859
*LHX6*	−0.5555	0.5738	0.4645–0.7087	0.0000

**Table 2:** Univariate and multivariate analyses of prognostic factors and overall survival of GBM patients.

Training Set	Univariate Cox Regression Analysis			Multivariate Cox Regression Analysis		
	HR	95% CI	*p*-Value	HR	95% CI	*p*-Value
Risk (high/low)	2.719	2.218–3.334	6.352 × 10^−22^	2.471	2.006–3.044	1.839 × 10^−17^
Age (≥60/<60)	1.866	1.544–2.255	1.093 × 10^−10^	1.583	1.304–1.922	3.341 × 10^−06^
Gender (male/female)	0.852	0.703–1.033	0.103	0.957	0.789–1.162	0.657
**Validation Set 1**						
Risk (high/low)	2.476	1.584–3.872	6.99 × 10^−05^	2.286	1.429–3.657	5.605 × 10^−04^
Age (≥60/<60)	1.796	1.061–3.041	0.029	1.365	0.788–2.364	0.267
Gender (male/female)	1.233	0.798–1.905	0.346	1.099	0.708–1.707	0.673
**Validation Set 2**						
Risk (high/low)	2.293	1.573–3.343	1.592 × 10^−05^	2.17	1.481–3.179	7.026 × 10^−05^
Age (≥60/<60)	2.449	1.734–3.459	3.637 × 10^−07^	2.35	1.655–3.337	1.785 × 10^−06^
Gender (male/female)	0.902	0.637–1.277	0.56	0.823	0.579–1.168	0.275

**Table 3:** The performance of our model and the models of existing four signatures.

Training Set	Our Model	The Model of Yin’s Signature	The Model of Pan’s Signature	The Model of Wang’s Signature	The Model of Cheng’s Signature
*p*-value (between low and high risk)	<0.0001	0.0012	0.0092	<0.0001	0.00046
AUC value of 1 year	0.7	0.59	0.53	0.67	0.57
AUC value of 2 years	0.79	0.64	0.57	0.68	0.61
AUC value of 3 years	0.86	0.63	0.57	0.7	0.66
**Validation Set 1**					
*p*-value (between low and high risk)	<0.0001	0.015	0.1	0.092	0.19
AUC value of 1 year	0.75	0.68	0.62	0.42	0.57
AUC value of 2 years	0.74	0.7	0.6	0.55	0.68
AUC value of 3 years	0.79	0.65	0.64	0.49	0.63
**Validation Set 2**					
*p*-value (between low and high risk)	<0.0001	0.00039	0.0013	0.0047	<0.0001
AUC value of 1 year	0.61	0.6	0.55	0.63	0.61
AUC value of 2 years	0.71	0.57	0.68	0.72	0.74
AUC value of 3 years	0.74	0.61	0.74	0.85	0.82

## Data Availability

The data sets used in this study can be downloaded from https://www.ncbi.nlm.nih.gov/geo/query/acc.cgi?acc=GSE4412(GSE4412), accessed on 20 August 2021, https://www.ncbi.nlm.nih.gov/geo/query/acc.cgi?acc=GSE4271(GSE4271), accessed on 20 August 2021, https://www.ncbi.nlm.nih.gov/geo/query/acc.cgi?acc=GSE16011(GSE16011), accessed on 20 August 2021, https://portal.gdc.cancer.gov/(TCGA), accessed on 20 August 2021, their published articles including additional files.

## Supplementary Materials

The following supporting information can be downloaded at: https://www.mdpi.com/article/10.3390/biomedicines10020317/s1, Table S1: Demographic and clinical characteristics of patients, Table S2: Differentially expressed genes, Table S3: Survival related genes, Table S4: Univariate and Multivariate analyses of clinical and molecular factors for overall survival of GBM patients in TCGA cohort and GSE16011 cohort, Figure S1: Comparisons of Kaplan-Meier analysis, ROC curves of different models in validation cohorts, Figure S2: Prognostic significance of clinical indicators and risk scores in the TCGA cohort, Figure S3: Prognostic significance of clinical indicators and risk scores of the GSE16011 cohort.
