# Supplementary material for: A Novel 16-Genes Signature Scoring System as Prognostic Model to Evaluate Survival Risk in Patients with Glioblastoma
Source: Biomedicines. 2022 Jan 29;10(2):317. doi: 10.3390/biomedicines10020317 (PMC8869708; doi:10.3390/biomedicines10020317)
Supplement: Supplementary file 1 [file biomedicines-10-00317-s001.zip › biomedicines-1518262-supplementary/Figure S1ú║ Comparisons of Kaplan-Meier analysis, ROC curves of different models in validation cohorts.pdf]

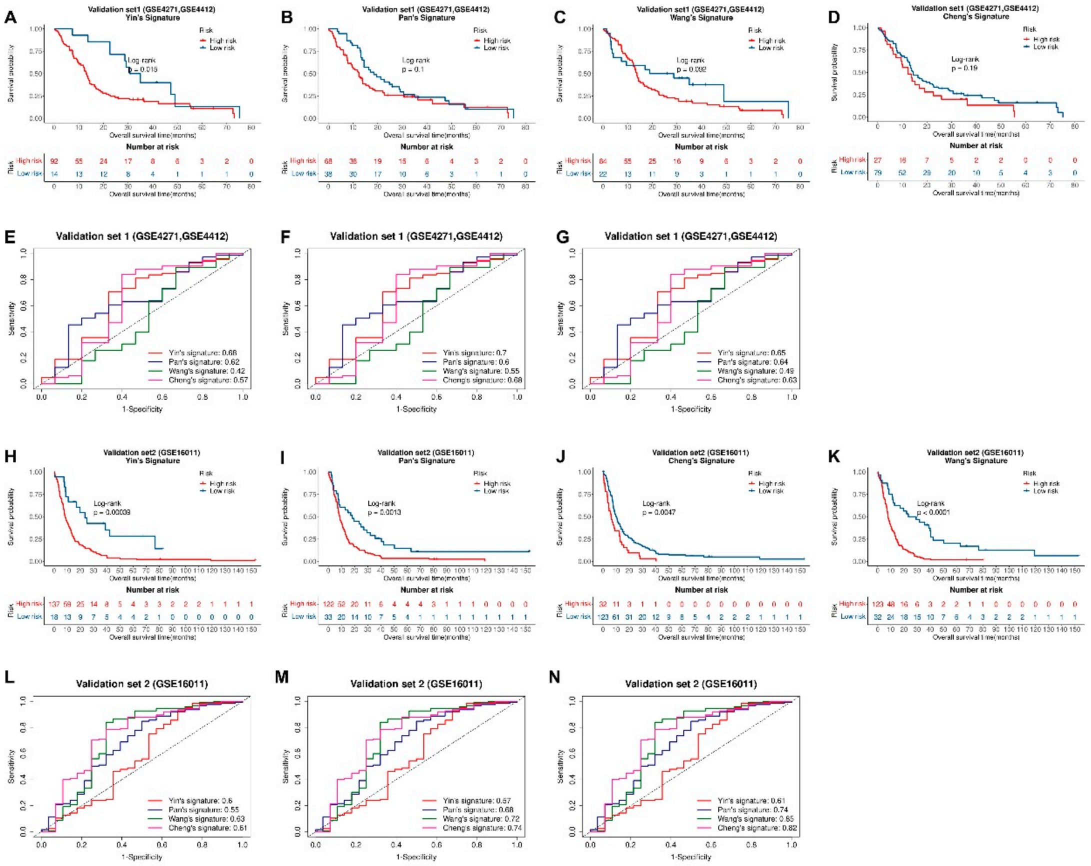

Figure S1. Comparisons of Kaplan-Meier analysis, ROC curves of different models in validation cohorts. (A-D) Kaplan-Meier analysis of different models in validation set1. (E-G) ROC curves of predicting 1-, 2-, 3-year overall survival in validation set1. (H-K) Kaplan-Meier analysis of different models in validation set2. (L-N) ROC curves of predicting 1-, 2-, 3-year overall survival in validation set2.
