# Supplementary material for: A Novel 16-Genes Signature Scoring System as Prognostic Model to Evaluate Survival Risk in Patients with Glioblastoma
Source: Biomedicines. 2022 Jan 29;10(2):317. doi: 10.3390/biomedicines10020317 (PMC8869708; doi:10.3390/biomedicines10020317)
Supplement: Supplementary file 1 [file biomedicines-10-00317-s001.zip › biomedicines-1518262-supplementary/Figure S2ú║ Prognostic significance of clinical indicators and risk scores in the TCGA cohort.pdf]

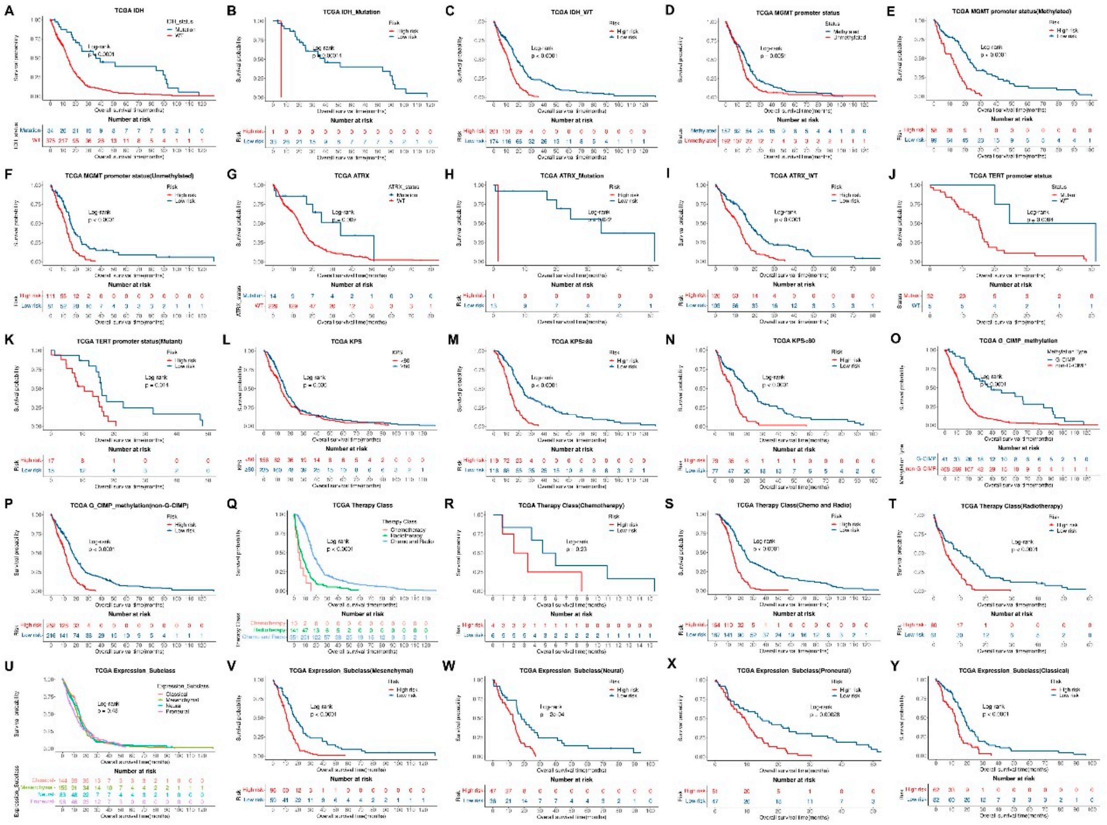

Figure S2. Prognostic significance of clinical indicators and risk scores in the TCGA cohort. (A)Kaplan-Meier overall survival analysis of GBM patients stratified by *IDH* status; (B-C) Kaplan-Meier overall survival analysis of high and low risk groups in combination with *IDH* status; (D) Kaplan-Meier overall survival analysis of GBM patients stratified by *MGMT* status only; (E-F) Kaplan-Meier overall survival analysis of high and low risk groups combined with *MGMT* status; (G)Kaplan-Meier overall survival of GBM patients stratified by *ATRX* status; (H-I) Kaplan-Meier overall survival analysis of high and low risk groups combined with *ATRX* status; (J) Kaplan-Meier overall survival analysis of GBM patients stratified by *TERT* status only; (K) Kaplan-Meier overall survival analysis of high and low risk groups combined with *TERT* status; (L) Kaplan-Meier overall survival analysis of GBM patients stratified by KPS score only; (M-N) Kaplan-Meier overall survival analysis of high and low risk groups combined with KPS; (O) Kaplan-Meier overall survival analysis of GBM patients stratified by methylation modification status only; (P) Kaplan-Meier overall survival analysis of high and low risk groups combined with methylation modification status; (Q) Kaplan-Meier overall survival analysis of GBM patients stratified by therapeutic modalities; (R-T) Kaplan-Meier overall survival analysis of high and low risk groups combined with therapeutic modalities; (U) Kaplan-Meier overall survival analysis of GBM patients stratified by expression subtypes; (V-Y) Kaplan-Meier overall survival analysis of high and low risk groups combined with expression subtypes.
