# Supplementary material for: A Novel 16-Genes Signature Scoring System as Prognostic Model to Evaluate Survival Risk in Patients with Glioblastoma
Source: Biomedicines. 2022 Jan 29;10(2):317. doi: 10.3390/biomedicines10020317 (PMC8869708; doi:10.3390/biomedicines10020317)
Supplement: Supplementary file 1 [file biomedicines-10-00317-s001.zip › biomedicines-1518262-supplementary/Figure S3ú║ Prognostic significance of clinical indicators and risk scores of the GSE16011 cohort.pdf]

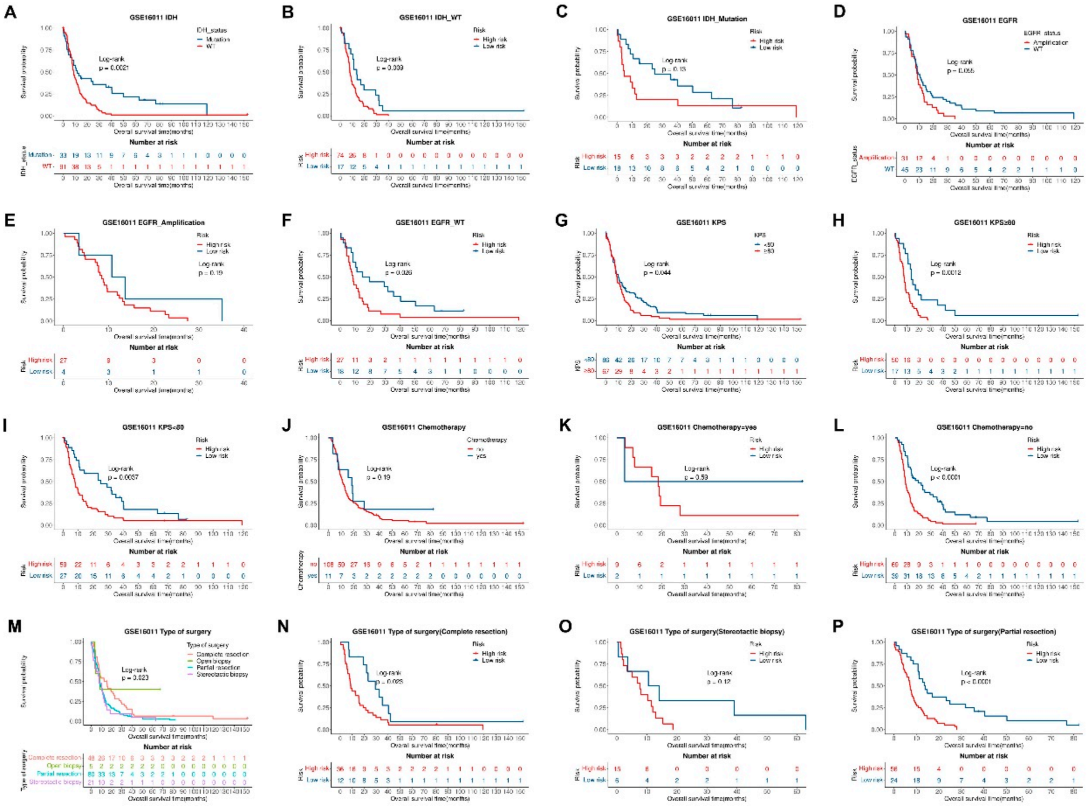

Figure S3. Prognostic significance of clinical indicators and risk scores of the GSE16011 cohort. (A) Kaplan-Meier overall survival analysis of GBM patients stratified by *IDH* status only; (B-C) Kaplan-Meier overall survival analysis of high and low risk groups combined with *IDH* status; (D) Kaplan-Meier overall survival analysis of GBM patients only stratified according to *EGFR* status; (E-F) Kaplan-Meier overall survival analysis of high and low risk groups combined with *EGFR* status; (G) Kaplan-Meier overall survival analysis of GBM patients stratified by KPS score only; (H-I) Kaplan-Meier overall survival analysis of high and low risk groups combined with KPS; (J) Kaplan-Meier overall survival analysis of GBM patients stratified by therapeutic modalities (with or without chemotherapy); (K-L) Kaplan-Meier overall survival analysis of high and low risk groups combined with therapeutic modalities (with or without chemotherapy); (M) Kaplan-Meier overall survival analysis of GBM patients only stratified according to type of surgery only; (N-P) Kaplan-Meier overall survival analysis of high and low risk groups combined with type of surgery.
