# Supplementary material for: A Novel 16-Genes Signature Scoring System as Prognostic Model to Evaluate Survival Risk in Patients with Glioblastoma
Source: Biomedicines. 2022 Jan 29;10(2):317. doi: 10.3390/biomedicines10020317 (PMC8869708; doi:10.3390/biomedicines10020317)
Supplement: Supplementary file 1 [file biomedicines-10-00317-s001.zip › biomedicines-1518262-supplementary/Table S1ú║Demographic and clinical characteristics of patients.pdf]

Table S1. Demographic and clinical characteristics of patients

Clinical indicators	Training set	Validation set 1		Validation set 2
	TCGA (N=524)	GSE4412 (N=50)	GES4271 (N=56)	GSE16011 (N=155)
**Age**, median (min~max)	59 (10~89)	49.5 (18~82)	49 (22~82)	55.2 (14.38~80.65)
**Gender**				
Male	320	23	39	105
Female	204	27	17	50
**Survival status**				
Dead	450	37	50	147
Alive	74	13	6	8
**IDH status**				
WT	375	-	-	91
Mut	34	-	-	33
NA	115	-	-	31
EGFR				
WT	-	-	-	45
Amp	-	-	-	31
NA	-	-	-	79
**1p/19q codeletion**				
Codel	2	-	-	-
Non-codel	502	-	-	-
NA	20	-	-	-
**MGMT Promoter**				
Meth	157	-	-	-
Unmeth	192	-	-	-
NA	175	-	-	-
**Expression Subclass**				
Classical	144	-	-	-
Mesenchymal	155	-	-	-
Neural	83	-	-	-
Proneural	98	-	-	-
NA	39	-	-	-
**TERT status**				
WT	5	-	-	-
Mut	32	-	-	-
NA	487	-	-	-
**ATRX status**				
WT	229	-	-	-

Mut	14	-	-	-
NA	281	-	-	-
**KPS**				
>=80	292	-	-	102
<80	99	-	-	51
NA	133			2
**Radiotherapy**		-	-	
Yes	141	-	-	120
NA		-	-	35
**Chemotherapy**				
Yes	10	-	-	11
No		-	-	108
NA		-	-	36
**Radio and Chemo**				
yes	351	-	-	-
NA	-	-	-	-
**Type of surgery**				
Complete resection	-	-	-	48
Open biopsy	-	-	-	5
Partial resection	-	-	-	80
Stereotactic biopsy	-	-	-	21
NA	-	-	-	1
