# Supplementary material for: A Novel 16-Genes Signature Scoring System as Prognostic Model to Evaluate Survival Risk in Patients with Glioblastoma
Source: Biomedicines. 2022 Jan 29;10(2):317. doi: 10.3390/biomedicines10020317 (PMC8869708; doi:10.3390/biomedicines10020317)
Supplement: Supplementary file 1 [file biomedicines-10-00317-s001.zip › biomedicines-1518262-supplementary/Table S4ú║Univariate and Multivariate analyses of clinical and molecular factors for overall survival.pdf]

Table S4. Univariate and Multivariate analyses of clinical and molecular factors for overall survival of GBM patients in TCGA cohort and GSE16011 cohort.

**Training set**	**Univariate Cox regression analysis**			**Multivariate Cox regression analysis**		
	**HR**	**95%CI**	***P*-Value**	**HR**	**95%CI**	***P*-Value**
Risk (high/low)	0.379	0.271-0.531	1.562×10^{-08}	0.337	0.236-0.482	2.74×10^{-09}
Age (≥60/<60)	1.564	1.146-2.135	4.816×10^{-03}	1.121	0.803-1.564	0.502
Gender (male/female)	0.578	0.419-0.798	8.54×10^{-04}	0.643	0.461-0.896	9.128×10^{-03}
IDH Status	0.431	0.06-3.096	0.403	0.738	0.099-5.494	0.767
MGMT promoter status	0.748	0.547-1.024	0.07	1.041	0.757-1.431	0.806
Expression Subclass	1.018	0.886-1.169	0.8	0.969	0.836-1.123	0.677
Therapy Class	2.67	1.996-3.572	3.648×10^{-11}	3.148	2.264-4.377	9.066×10^{-12}
KPS Score (<80/≥80)	0.721	0.529-0.983	0.038	0.717	0.521-0.989	0.042
**validation set 2**						
Risk (high/low)	2.417	1.494-3.911	3.237×10^{-04}	2.383	1.341-4.234	3.082×10^{-03}
Age (≥60/<60)	2.495	1.529-4.071	2.533×10^{-04}	2.209	1.328-3.673	2.257×10^{-03}
Gender (male/female)	1.302	0.811-2.091	0.274	1.275	0.768-2.116	0.347
IDH Status	0.381	0.219-0.663	6.372×10^{-04}	0.538	0.274-1.056	0.071
Type of surgery	1.171	0.948-1.446	0.143	1.401	1.108-1.771	4.845×10^{-03}
Chemotherapy (Yes/No)	1.75	0.839-3.652	0.136	2.618	1.209-5.669	1.459×10^{-02}
KPS score (<80/≥80)	0.502	0.308-0.818	5.675×10^{-03}	0.516	0.303-0.88	0.015

TCGA cohort (206 patients) and GSE16011 cohort (92 patients)
